# Simulation Study on the Optimisation of Replenishment of Landscape Water with Reclaimed Water Based on Transparency

**DOI:** 10.3390/ijerph20054141

**Published:** 2023-02-25

**Authors:** Dong Ao, Lijie Wei, Liang Pei, Chengguo Liu, Liming Wang

**Affiliations:** 1 College of Environmental and Chemical Engineering, Xi’an Polytechnic University, Xi’an 710048, China; 2 Xinjiang Institute of Ecology and Geography, Chinese Academy of Sciences, Urumqi 830011, China; 3 Institute of Geographic Sciences and Natural Resources Research, Chinese Academy of Sciences, Beijing 100101, China; 4 China National Chemical Urban Investment Company Limited, Xi’an 710048, China

**Keywords:** landscape urban water, water transparency, algae growth, water replenishment, reclaimed water

## Abstract

Water-scarce cities have fewer surface water (SW) resources available for ecological use, causing landscape water to deteriorate due to water shortage and fail to perform their intended landscape functions. As a result, many cities use reclaimed water (RW) to replenish them. However, this could cause concern among the people, as RW usually has higher nutrient concentrations, which may stimulate algae growth and deteriorate the aesthetic senses of the receiving water bodies. In order to assess the feasibility of using RW for this purpose, this study used Xingqing Lake in Northwest China as insight into the effect of RW replenishment on the visual landscape quality of urban landscape water. Water transparency (measured by SD) is used as an intuitive indicator to reflect the comprehensive influence of suspended solids and algae growth on the water’s aesthetic quality. Scenario analyses were carried out after calibrating and validating one-year data in MIKE 3 software with both SD and algae growth calculations, and the results showed that the low concentration of suspended matter in RW could compensate for the decrease in SD due to algal blooms caused by high concentrations of nitrogen and phosphorus, and the effect on SD is especially pronounced under conditions that are not conducive to algal growth, such as good flow conditions and low temperature. In addition, to meet a SD ≥ 70 mm, the total water inflow required can be significantly reduced with the optimal application of RW. It is also indicated that partial or complete utilization of RW to replace SW for replenishing the landscape water could be feasible from the viewpoint of landscape quality, at least for the landscape water investigated in this study. This can provide a method for the improvement to urban water management practices by using RW for replenishment in water-scarce cities.

## 1. Introduction

Due to economic development and population growth, a decreasing volume of surface water (SW) is available to replenish urban lakes and ponds, especially in water-scarce areas. At the same time, in order to improve people’s quality of life, more and more artificial urban landscape waters have rapidly emerged. However, with the growing imbalance between urban water supply and demand, the available sources of SW for the supplementation of urban water bodies have gradually declined. This has resulted in deterioration of the water quality of urban waters and even led to the formation of black, malodorous water bodies, which seriously impact the landscape quality of lakes and ponds as recreational areas for residents [1,2]. Therefore, it is necessary to find alternative water sources as a supplementary water source for urban waters. Reclaimed water (RW) from wastewater treatment plants (WWTPs) effluents, especially for those mainly treating the residential domestic wastewater, provides a stable quantity and quality of water resources. Additionally, its reuse involves low operating costs [3,4,5]. Thus, many cities use RW as a source of replenishment water for urban waters.

Using RW can solve the water shortage problem of urban waters replenishment, but the quality of RW is significantly different from that of SW and, thus, may have a great impact on the quality of the receiving water body. Current researchers are mostly concerned about the distribution and impact of nutrients, heavy metals, trace organic pollutants, and pathogens of urban waters replenished with RW [5,6,7]. The research indicated that heavy metal concentration and ecotoxicity did not differ significantly between the urban water replenished with RW and SW [7,8]. By contrast, significant differences were observed in algal growth and the pathogen risk [6,7]. In fact, urban waters have many functions, including maintaining the diversity of the water ecosystem, flood resistance, and providing a good living landscape environment. However, perhaps what the residents are more aware of and most want is to maintain a better water aesthetic quality, especially for landscape water [4]. That can be represented by many indicators, including color, turbidity, algae quantity, and water transparency [9]. Among these indicators, chromaticity and turbidity are caused only by dissolved and insoluble substances in a water body, respectively. However, transparency can comprehensively reflect the turbidity, color, and the abundance of algae in the water body, and it is also one of the most intuitive indicators for people to use to evaluate the quality of urban waters [10,11]. Transparency is significantly positively correlated with public satisfaction surveys, which is based on the results of the research group’s previous survey on 189 urban water bodies [12]. Moreover, water transparency is also an important factor reflecting the survival of aquatic organisms and pathogen risks in water bodies [13,14]. In addition, in many comprehensive landscape indicators, transparency has a higher proportion in them [9,15]. Therefore, water transparency is an important indicator to represent the visual landscape environment and comprehensive water quality of water bodies. More importantly, among these indicators, transparency can be calculated and expressed through a series of formulas according to its calculation principle, which is conducive to some problems that are not suitable for experimentation and can be solved with water quality models.

As motioned, the quality of RW and SW are quite different, and the effects on water transparency of landscape water replenished with RW are different from those of SW. Compared with SW, the nutrient concentration in RW is high, but RW has a low suspended solids (SS) concentration due to the sedimentation and filtration processes of wastewater treatment plants. Water transparency refers to the depth of light transmission in water and is essentially the attenuation of light in water due to both absorption and scattering by substances in a water body. It is related to the algal population in a water body, as well as many other factors, such as inorganic suspended solids (ISS) [16,17,18]. Therefore, when RW is used as a source of water for replenishment, the lower concentration of SS could be conducive to the increase in transparency. In contrast, the high concentration of nutrients such as nitrogen and phosphorus of RW, which may promote the growth of phytoplankton, result in an increase in the abundance ofalgae, thereby reducing the transparency of the water body. Thus, the effects of urban water replenishment with RW on the water transparency remain unclear. Moreover, the amount of algae depends not only on the water quality of the inflow; it also depends on the total amount of water replenished (i.e., the frequency of water changes in the waters or the dilution effect), as well as the external environmental conditions such as temperature, light, etc. [19,20,21]. Of course, the concentration of phytoplankton and ISS could be reduced by increasing the total water flow, which mainly contributes to increasing the frequency of water change or dilution, thereby increasing the transparency of the water body. In addition, as algae growth is also affected by the external environment conditions. When it is not suitable for algae growth, although the concentration of nutrients will increase in the water body due to the increased proportion of RW in its replenishment water source, the amount of algae will not increase rapidly, as the environment is not suitable; thus, transparency should be improved by its lower SS concentration compared to SW. Conversely, when it is suitable for algae growth, it may be necessary to reduce the RW proportion to ensure the transparency of the water body. In other words, in order to maintain the transparency of urban water bodies, in addition to increasing the total water replenishment, the RW proportion can be adjusted by optimizing the water replenishment scheme to reduce the total water inflow required. Perhaps this is a better way for water-scarce regions to use more water elsewhere and ease water stress in cities. Therefore, the overall effect of urban water replenishment with RW on the water quality and water transparency needs to be studied.

Nonetheless, as the water quality of actual water bodies is easily disturbed by the external factors, it is difficult to study the effects of RW replenishment on urban waters through small-scale experimental methods in the laboratory [22]. The water quality model is a numerical representation of the physical, chemical, and biological transformation processes and can reflect changes in the actual water quality through the calibrations of the water quality model; thus, it is suitable for analyzing these practical problems. Of the many models available to study real water bodies, MIKE software is an excellent comprehensive model with a wide range of applications, including the simulation of the water quality in lakes, estuaries, coastal areas, and other water bodies [23,24].

The objective of this research was to study the impact of RW replenishment on the quality and transparency of urban landscape water and to assess the feasibility of using RW from the viewpoint of landscape quality control. This was done by establishing a water transparency calculation model in MIKE 3 software based on an existing detailed, physically based water quality model. A scenario analysis was carried out using an urban landscape water in Northwest China, which looked at the constraints of its surface water sources and the demand for RW replenishment. Based on this simulation, a water supply scheme of RW replenishment for the water body is proposed, with water transparency as the control indicator. The results provide a solution to the problem of poor landscape water quality due to low water replenishment in water-scarce areas.

## 2. Materials and Methods

### 2.1. Case Study Introduction and Water Quality Data Collection

#### 2.1.1. Lake Introduction

Xingqing Lake is a typical example of an urban landscape waters in the city of Xi’an in Northwest China, which is located in Xingqing Park. Xingqing Park covers an area of 52 hectares, and the lake has a surface area of 10 hectares. It has a depth of 1.5–2.0 m and a storage capacity of up to 200,000 m^3^. It is maintained by SW transported via laid artificial pipes from a river in Xi’an City, and its maximum designed intake is 0.77 m^3^/s. As Xi’an has a serious water shortage, the water intake in previous years was 0.05–0.5 m^3^/s, with an average annual intake of less than 0.1 m^3^/s. Its long hydraulic retention time has led to poor water quality, and the transparency of the water body is even below 0.30 m [25], which seriously impacts the public’s opinion of the waterscape. The municipality is therefore prepared to use RW to solve its replenishment problem.

#### 2.1.2. Sampling and Water Quality Analysis

**Figure 1:**
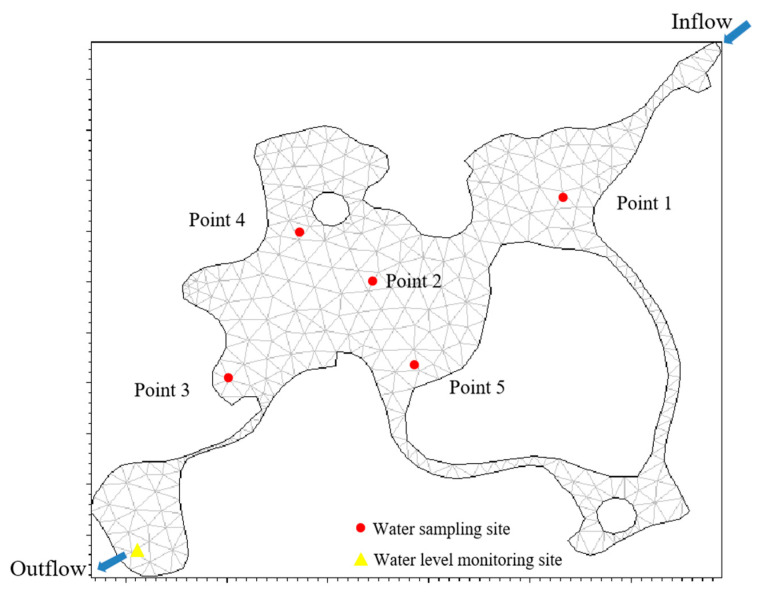
Water quality sampling points (including the inflow) and model grid of Xingqing Lake.

The water quality of Xingqing Lake was evaluated from January to December at five sampling points (except the inflow), as shown in Figure 1. The frequency of sampling was once a month between 10 and 11a.m. on the 1st and 10th day of each month, avoiding rainy days. Water samples were taken from 0.5 m below the water surface at each point, with three liters of water taken at each sampling point. After collecting the samples, they were preserved with ice, immediately transported back to the laboratory, and processed and tested as soon as possible. Water temperature, dissolved oxygen, and transparency were measured onsite. Transparency was measured by using a Secchi disk (abbreviated as SD). Water temperature and dissolved oxygen were measured using a WTW Multi 3410 portable multiparameter water quality meter.

One liter unfiltered water samples were taken to determine the total nitrogen (TN), total phosphorus (TP), and chlorophyll a (Chl-a). The rest of the water samples were used to determine the ISS and dissolved nitrate nitrogen (NO_3_^−^-N), ammonia nitrogen (NH_4_^+^-N), and inorganic phosphorus (IP). The ISS were measured using membrane filtration, whereby ISS were first filtered and then dried in a muffled oven to remove the organic matter for weight measurement. The TN was measured by ultraviolet spectrophotometry, NH_4_^+^-N by Nessler’s reagent spectrophotometry, and NO_3_^−^-N by the spectrophotometric method with phenol disulfonic acid. The TP and IP were measured by the ammonium molybdate spectrophotometric method. Chl-a was extracted with 90% acetone and measured by the spectrophotometric method. The concentration of Chl-a was calculated by the equation of 11.64 OD_663_ − 2.16 OD_645_ + 0.1(OD_630_ − OD_750_). The detail processes of those indices were according to the standard methods for the examination of water and wastewater [26].

#### 2.1.3. Lake Replenishment Water Quality

For the simulation of the SW and RW quality of lake replenishment sources, we used the five-category water quality standard for the SW quality (with SS concentrations from the SL63-94 surface water resource quality standard) [27] and RW landscape reuse standards [28]. As the nitrogen and phosphorus indicators in the standard are NH_4_^+^-N, TN, and TP, the nitrogen and phosphorus indicators used for the variables in the eutrophication module were NH_4_^+^-N, NO_3_^−^-N, and IP. To unify the quality of RW and SW, it was assumed that the NO_3_^−^-N in the scenario analysis was the difference between the TN and NH_4_^+^-N for both and that the TP was all IP. The water quality indicators for SW and RW are shown in Table 1.

### 2.2. Method for Evaluating Water Transparency

SD is calculated using the relational equation established by Tyler and Preisendorfer [29], which indicated that SD is mainly based on the ability of the human eye to detect contrast and the attenuation of light caused by the optical properties of the water. SD is influenced by inorganic suspended particles, phytoplankton, and colored dissolved organic matter (CDOM). The specific relationship is as follows:

(1) $$\mathrm{SD}=\gamma /\left(c\left(\lambda \right)+K_{d}\right)$$

where SD is the water transparency, γ is a function of the eye’s contrast threshold (a coupling constant, 8.9 m), and c(λ) and *K_d_* are the depth-averaged beam and downwelling irradiance attenuation coefficients, respectively. c(λ) can be expressed as:

(2) c(λ)=a(λ)+b(λ)

where a(λ) and b(λ) are the total absorption and scattering coefficients of the water body, respectively.

a(λ) and b(λ) are obtained by multiplying the coefficients of each influencing substance by its concentration. Since the attenuation of light in the visible spectrum caused by CDOM is much weaker than SS, including algae and non-algal suspended matter [30], it was not considered here. In addition, as the attenuation coefficients of organic non-algal suspended matter and ISS differ largely, non-algal suspended material was divided into those two parts [31,32]. The main source of organic non-algal suspended matter in the water was organic detritus, which is expressed as detritus carbon (DC) in the model [31,33]. ISS was assumed to not be affected by the biological and chemical transformations in the water, and it was mainly from the water source. Thus, the formulae for calculating a(λ) and b(λ) are as follows:

(3) $$a\left(\lambda \right)=a_{w}\left(\lambda \right)+a_{\varphi }\left(\lambda \right)\cdot \left[\mathrm{CHL}\right]+a_{p-\varphi }\left(\lambda \right)\cdot \left[\mathrm{ISS}\right]+a_{p-\varphi }^{*}\left(\lambda \right)\cdot \left[\mathrm{DC}\right]$$

(4) $$b\left(\lambda \right)=b_{w}\left(\lambda \right)+b_{\varphi }\left(\lambda \right)\cdot \left[\mathrm{CHL}\right]+b_{p-\varphi }\left(\lambda \right)\cdot \left[\mathrm{ISS}\right]+b_{p-\varphi }^{*}\left(\lambda \right)\cdot \left[\mathrm{DC}\right]$$

where λ is the wavelength, a_w_(λ) and b_w_(λ) are the absorption and scatting of pure water (1/m), a_φ_(λ) and b_φ_(λ) are the chlorophyll-specific absorption and scatting coefficients (m^2^/mg Chl-a), a_(p−φ)_(λ) and b_(p−φ)_(λ) are the absorption and scatting coefficient of ISS (m^2^/g ISS), and a_(p−φ)_(λ) and b_(p−φ)_(λ) are the absorption and scatting coefficients of detritus carbon (m^2^/g DC), respectively. CHL, ISS, and DC represent the concentrations of Chl-a (µg/L), ISS (mg/L), and DC (mg/L), respectively. 

The relation between *K_d_*, a(λ), and b(λ) in the photic zone was according to Kirk [34], which is expressed as:

(5) $$K_{d}=a\left(\lambda \right)\left[\frac{{\left(1+\left(0.425\times \mu_{0}-0.19\right)\times \frac{b\left(\lambda \right)}{a\left(\lambda \right)}\right)}^{0.5}}{\mu_{0}}\right]$$

where *μ*_0_ is the average cosine of the refracted solar angle (approximately constant).

As the equation for calculating SD is related to the concentrations of Chl-a, DC, ISS, and organic suspended solids in the water, based on this, ECO Lab 2 was chosen as the eutrophication module for the water. The state variables and the exact equations for other processes can be found in the “ECO Lab Template-Scientific Description” [35]. The specific procedures of embedding can be found in the authors’ previous publications [17].

### 2.3. Water Quality Modeling Process 

#### 2.3.1. Mesh Generation

A full-water simulation of Xingqing Lake was conducted, with the coordinates of the lake shore points obtained from Baidu Maps. Data was fed into the model, and the boundary nodes were redistributed and boundaries smoothed to create the water–land boundary for the body of water.

Considering the topographic characteristics of this water body, a triangular grid was used to generalize the grid of Xingqing Lake’s water area to a total of 588 grids with a minimum angle of 26.4°.

For vertical stratification, the Sigma stratification method was used to divide the water depth into three layers. As the discharge from the lake drains based on height adjustments to its sluices, its discharge was managed by means of a fixed water level. The calculation grid is shown in Figure 1.

#### 2.3.2. Boundary Condition Setting

The hydrodynamic model boundary conditions are the daily average value inflow provided by the park administration and the outflow controlled by the sluice height. Hourly rainfall and wind speed and direction, as well as the boundary conditions of temperature and salinity, including hourly air temperature, atmospheric pressure, and solar light intensity, were obtained from actual data measured by the municipal meteorological bureau. Rainfall runoff from the water body, which only considers runoff caused by impermeable paved surfaces, covers an area of approximately 0.067 km^2^. Runoff volumes were calculated using the runoff coefficient, with specific runoff coefficients derived from data in the literature [36].

The water quality module boundary conditions include point source pollution and non-point source pollution. Point source pollution comes from the replenishment water, and the water quality was measured. The main indicators are shown in Table 2. As can be seen from Table 2, during the rainy periods of early summer and early autumn, the nutrient concentration in the influent is lower due to the washing of rain, while the SS is higher. Sources of non-point pollution were those from atmospheric dry deposition and surface runoff. The pollutants are mainly nitrogen and phosphorus nutrients. The pollution load of the dry deposition was estimated using the amount of dust fall and the concentration of pollutants. The monthly amounts of pollutants entering the lake were determined according to the results reported by Yun [37], in which the measured point is only about 1 km from the lake, the data are shown in Table 2, with the daily coefficient of variation each month regarded as 0. Pollution loads from the rainfall runoff were calculated from runoff volumes and runoff pollutant concentrations, which were then converted to wet deposition in Xingqing Lake. The concentrations of the runoff pollutants were as follows: 2.5 mg/L for NH_4_^+^-N, 4 mg/L for NO_3_^−^-N, and 0.64 mg/L for IP [38].

#### 2.3.3. Model Calibration and Validation

Based on the number of grids in the lake, the time step for the model calculation was set at 30 min, and the output time step for the simulation results was 90 min. As the water level of Xingqing Lake is determined by the height of the sluice, which remains essentially constant, only water temperature was used as a state variable for the parameter calibrations of the hydrodynamic model in this paper. The water quality model was then calibrated and validated using measured water quality data (including Chl-a, SD, NO_3_^−^-N, NH_4_^+^-N, and IP) from the sampling sites. In order to better illustrate the feasibility of the model, the model was first calibrated using measured data from January to July and then validated using measured data from August to December.

The model accuracy and precision are expressed in terms of Root mean square error (*RMSE*) and Nash–Sutcliffe efficiency coefficient (*NSE*) of the simulated and measured values, which are calculated as follows:

(6) $$RMSE=\sqrt{\sum_{i=1}^{n}{\left(Y_{i}-Y_{si}\right)}^{2}/n}$$

(7) $$NSE=1-\frac{\sum_{i=1}^{n}{\left(Y_{i}-Y_{si}\right)}^{2}}{\sum_{i=1}^{n}{\left(Y_{i}-\bar{Y}\right)}^{2}}$$

where *Y_i_* represents the measured value, *Y_si_* represents the simulated value, $\bar{Y}$ represents the mean of measured values, and *n* is the number of measurements. The *RMSE* is between 0 and ∞, and the smaller the *RMSE* value, better the precision of the model. The *NSE* is between −∞ and 1, and high values indicate the high accuracy of the model.

## 3. Results and Discussion

### 3.1. Model Calibration and Validation

#### 3.1.1. Hydrodynamic Model

**Figure 2:**
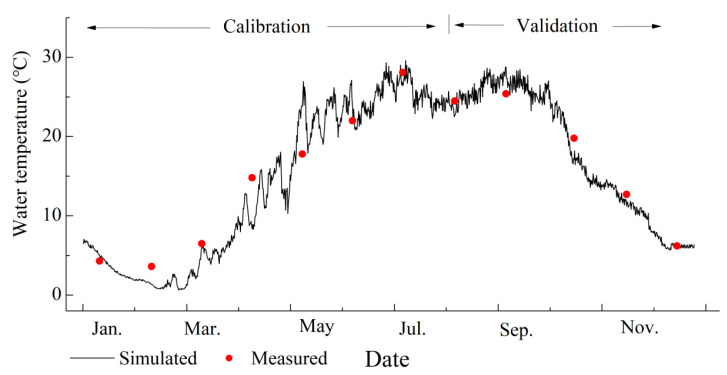
Simulated and measured values of the water temperature during the calibration and validation phases.

Through one-year data of the water level and water temperature, and after several adjustments to the model’s parameters, the values of the calibrated parameters of the hydrodynamic module were determined (Table 3). The results of the calibration and validation of the water temperature are shown in Figure 2 (the water level is not shown, as it is almost constant). During the calibration phase (January–July), the *RMSEs* between the simulated and measured data for the water level and water temperature were calculated to be 0.15 m and 1.76 °C, respectively. The corresponding *NSE* values were 0.87 and 0.89, respectively. The *RMSEs* for the water level and water temperature was 0.12 m and 1.37 °C, respectively, during the validation phase (August–December), and the *NSEs* were 0.88 and 0.91, respectively.

#### 3.1.2. Water Quality Model

**Figure 3:**
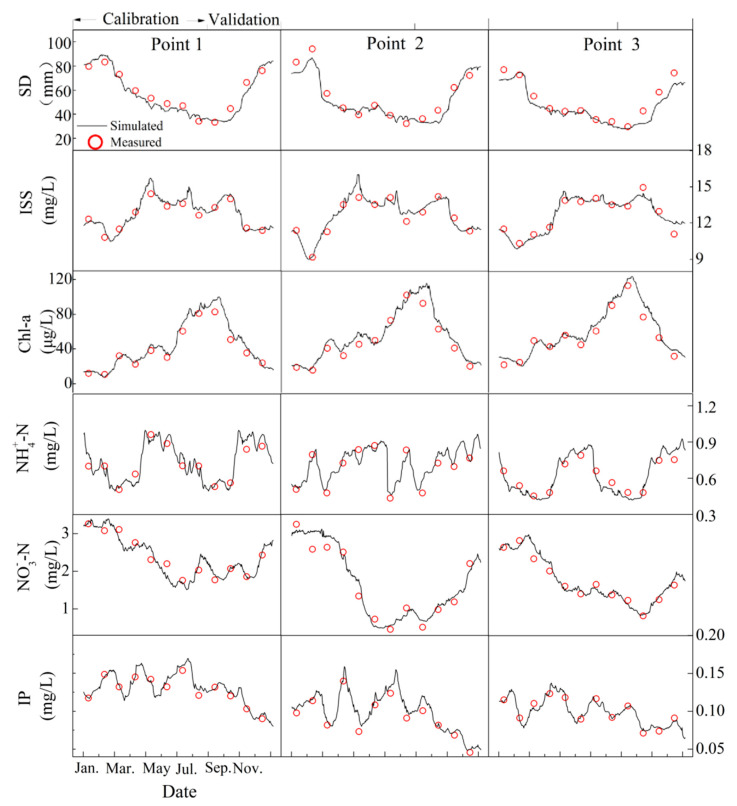
Simulated and measured values of the water quality in the calibration and validation phases.

Using the one-year water quality monitoring data from three of the sample points (Points 1–3), the model parameters were adjusted repeatedly to determine the values of the calibrated parameters for the main indicators (including SD, Chl-a, ISS, NH_4_^+^-N, NO_3_^−^-N, and IP) of the water quality model (Table 3). The key water quality results from the water quality model calibration and validation are shown in Figure 3. 

The *RMSEs* of the SD, ISS, Chl-a, NH_4_^+^-N, NO_3_^−^-N, and IP in the three points were calculated to be less than 7.31 mm, 2.04 mg/L, 7.12 μg/L, 0.08 mg/L, 0.30 mg/L, and 0.06 mg/L, respectively, during the calibration phase (January–July), and the corresponding *NSEs* were greater than 0.78, 0.82, 0.74, 0.76, 0.75, and 0.78, respectively. The *RMSEs* for SD, ISS, Chl-a, NH_4_^+^-N, NO_3_^−^-N, and IP were less than 7.61 mm, 2.36 mg/L, 6.45 μg/L, 0.10 mg/L, 0.25 mg/L, and 0.05 mg/L, respectively, during the validation phase (August to December), and their corresponding *NSEs* were 0.76, 0.80, 0.76, 0.74, 0.78, and 0.80, respectively. It can be seen from the results that the values of the *NSEs* are all higher than 0.74 (higher than 0.7 usually indicates that the calibration parameters of the model are reliable [39]); thus, the model calibrated in this case is valid and reliable for the simulation of the hydrodynamics, water quality, and transparency.

### 3.2. Model Simulations

#### 3.2.1. Changes in Water Quality under Various Replenishment Conditions

**Figure 4:**
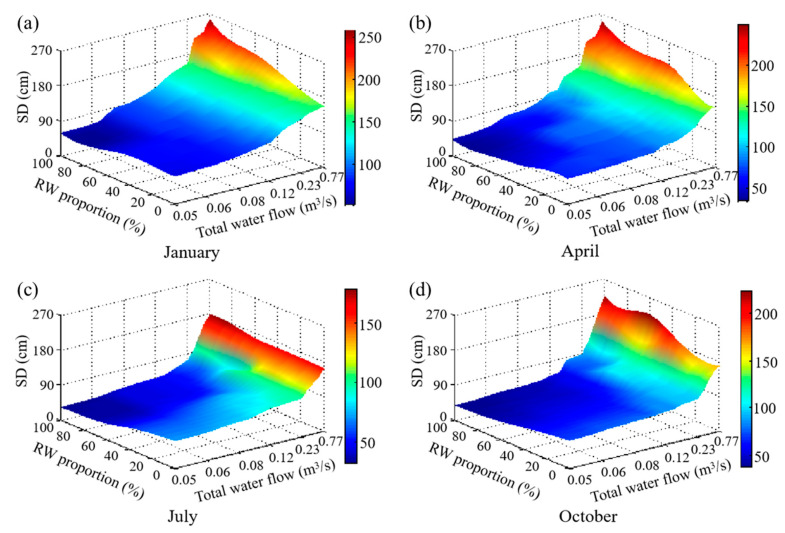
Simulated mean values of SD under different water replenishment qualities and quantities. (**a**) January, (**b**) April, (**c**) July and (**d**) October.

**Figure 5:**
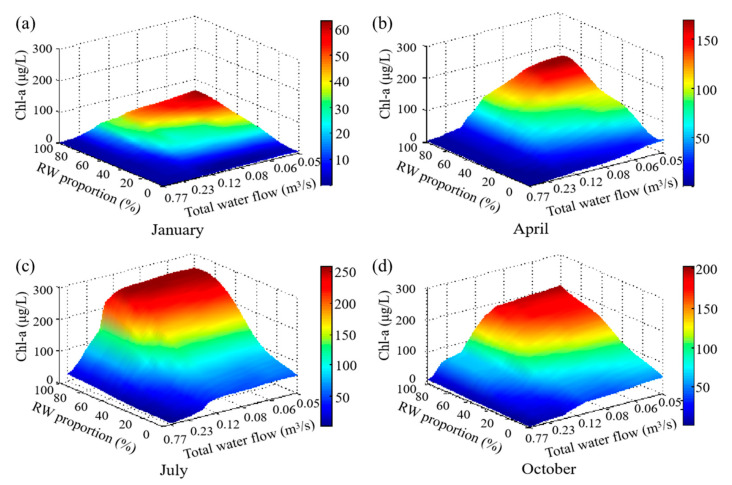
Simulated mean values of Chl-a under different water replenishment qualities and quantities. (**a**) January, (**b**) April, (**c**) July and (**d**) October.

After the model parameters were calibrated, the model was used to simulate water quality changes in Xingqing Lake under various scenarios. The inflow of the hydrodynamic model boundary conditions was the data of the total water inflow, and the meteorological conditions of that were to retain the actual data measured by the Municipal Meteorological Bureau. Since the SD is mainly related to the ISS, organic suspended solids, and algae quantity, and the algae quantity not only affects by the lake water quality but also, by the total water replenishment and external environment (temperature, light, etc.), the changes of the water quality under different total water replenishment, influent water quality (realized by adjusting the relative proportions of RW used for replenishment), and months were studied. In addition, the concentration of ISS in the water body is mainly affected by its concentration in the influent water, and most organic suspended solids in the water were mainly from the remains of dead algae, which were significantly related to Chl-a. Thus, it was not necessary to discuss it separately, and only the changes in algal abundance (Chl-a) were considered regarding the causes of landscape water quality changes. The mean values of the SD and Chl-a in Xingqing Lake simulated by the model for different months with different replenishment conditions (RW proportion and total water flow) are shown in Figure 4 and Figure 5, respectively.

As can be seen from Figure 4, when the quality of the replenishing water is certain (RW proportion fixed), the SD decreases as the replenishment decreases, while Chl-a has an S-shaped curve increasing trend. The magnitude of both the decrease in SD and the increase in Chl-a are related to the total water flow. This is because, when the water quality is fixed, the ISS concentration is little related to the quantity of water replenished. Therefore, the SD is mainly related to the algae quantity. When the total water flow decreases, the hydrodynamic conditions of the lake are poor, and the dilution effect is also weakened, which would stimulate algae proliferation and also influence the water transparency. In July, for example, when the RW proportion was fixed at 40%, the total water flow decreased from 0.77 m^3^/s to 0.46 m^3^/s, and the SD fell gradually from 162.93 cm to 144.76 cm. This was due to the wash-out of algae from the lake before they were mature, causing a slow increase in algae from 8 μg/L to 26 μg/L (Figure 5). However, the total water flow decreased from 0.29 m^3^/s to 0.13 m^3^/s, and the SD decreased rapidly and markedly from 144.76 to 77.24 cm, as the hydraulic retention time was conducive to algal growth, and there was plenty of nutrient replenishment at this time, with Chl-a increasing rapidly from 35 μg/L to 112 μg/L (Figure 5). In contrast, when the total water flow decreased from 0.13 m^3^/s to 0.05 m^3^/s, the SD fluctuated within a narrow range (44.51–67.24 cm). This was due to the low level of water replenishment and lack of nutrient replenishment despite high nutrient concentrations in the inflow water source [8,20], resulting in a high but stable algal population (112–124 μg/L). Furthermore, the trends in SD and Chl-a were consistent with the percentage of RW in different seasons.

On the other hand, the SD of the water bodies influenced by the RW proportion, creating different trends depending on the total water flow and the season (Figure 4). When the total water flow exceeds a certain limit, the SD increases with the increasing RW proportion. As the total water inflow was higher than 0.12 m^3^/s in April, Chl-a was always at a very low level. This is because, when the volume of water replenishment is higher, algae growth can be greatly inhibited [20,21], so Chl-a was always at a very low level. However, the corresponding ISS concentration of the total inflow deceased from 45 to 10 mg/L when the RW proportion increased from 0% to 100%. Thus, the increased RW proportion will reduce its ISS and increase the SD of the water bodies. Furthermore, algae growth is not only related to the nutrient concentration and the total water flow but also related to the environmental conditions, which means that the limit of the total water flow described above is related to the external environmental factors. When the environment conditions, such as temperature, are more suitable for algae growth, the number of algae will increase rapidly with the increase of the nutrient concentration by replenishing with RW, so it is necessary to increase the total water inflow (that is, the limit of the above total water flow is increased) to maintain the SD by inhibiting the algae growth and washing-out of algae. However, when it is not conducive to algae growth, such as a low temperature, it is not needed to greatly increase the total water inflow. For example, in January, April, October, and July, the replenishments of those were 0.05 m^3^/s, 0.12 m^3^/s, 0.23 m^3^/s, and 0.46 m^3^/s, respectively. However, when the total water flow is below these limits, the growth of algae is greatly affected by the external environment conditions, such as water temperature and light, and the trend of the SD with the RW proportion will vary depending on the month.

In general, in the spring and winter, when the temperature is low, the SD increases with the increase of the RW proportion. This is attributed to the fact that the algae growth is inhibited with the low temperature. The algae growth is less sensitive to the influence of the nutrient concentration and water inflow; thus, the SD is mainly related to the ISS concentration in the water body, and the higher the RW proportion, the lower the ISS concentration, so that the SD and RW proportion are positively correlated. However, in the summer and autumn, when temperatures are higher, there is no clear pattern. For example, in July, the total water flow is between 0.19 m^3^/s and 0.46 m^3^/s, and there are two peaks in the SD as the RW proportion increases at around 25% and 100% RW, respectively. When it is below 0.19 m^3^/s, the SD decreases while the RW proportion increases. The main reason for these results is that, under these conditions, algae growth is affected by a combination of factors such as nutrient concentration, nutrients, water flow velocity, and the diluting effect of water change in the water body [20,21,40].

#### 3.2.2. Water Replenishment Optimization

From the previous content analysis, it can be seen that Chl-a remained at a low level under the conditions of low temperature or high total water replenishment, and the SD increased with the increase of the proportion of RW with low suspended solids characteristics. Otherwise, the high nitrogen and phosphorus of the RW may cause algae growth to have a negative impact on the landscape water quality—that is, the SD shows a slow increase first, then a rapid decrease and then a slow increase, and even a trend of continuous decrease in the SD while the RW proportion increased. Therefore, the characteristics of low SS in the RW can be used to reduce the total water inflow required by optimizing the water replenishment scheme.

**Figure 6:**
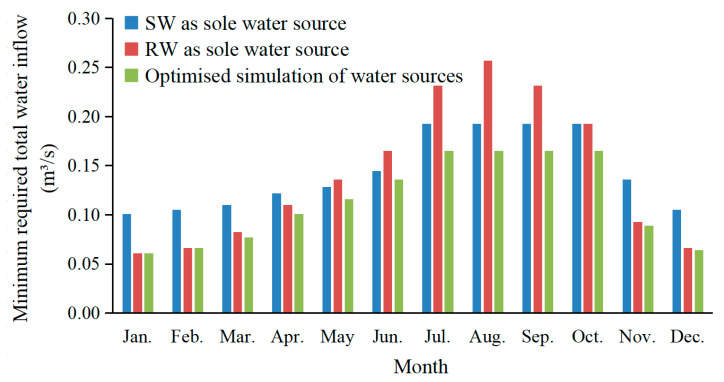
Minimum monthly total replenishment required for SD ≥ 70 cm under different replenishment conditions.

Taking the landscape water quality control target of SD ≥ 70 cm as an example for analysis, the corresponding minimum amount of the total water inflow required per month for Xingqing Lake under different water supply schemes is shown in Figure 6, which is according to the results of the model simulation after stabilization. As can be seen from the figure, in order to meet the landscape water quality requirements, as the sole source for water replenishment, the flow of the RW required is higher than that of the SW during the months of May to September, while, for rest of the year, the opposite is true. This is mainly due to the high temperatures in May–September, which are suitable for algae growth, and coupled with the high concentrations of nitrogen and phosphorus in the RW, the algae growth is faster, resulting in low SD [6,17]. Thus, there is a need to increase the amount of water replenishment and use the washing and dilution of water bodies to reduce the concentration of algae to meet the requirements of the landscape water quality. However, the temperature is comparatively lower in October–April, and the algae growth is inhibited, even if the nutrient concentrations are high in the RW replenishment. At this time, the SD is mainly affected by the ISS. Thus, it is conducive to the improvement of the SD with a higher proportion of the RW with the characteristics of low SS. This also means that it can be replenished with less water flow to meet the SD control criteria. Overall, it also suggests that a low ISS of the RW can compensate to an extent for the impact of algae growth on the SD caused by its high concentration of nutrients.

Importantly, it was found that the optimized water supply scheme (the minimum total water inflow required) was 75–100% RW in November–April and 25–35% RW in May–October for water replenishment (Figure 6). To meet the target of SD ≥ 70 cm, the minimum total water inflow required from January to December was 0.06 m^3^/s, 0.07 m^3^/s, 0.08 m^3^/s, 0.10 m^3^/s, 0.12 m^3^/s, 0.14 m^3^/s, 0.17 m^3^/s, 0.17 m^3^/s, 0.17 m^3^/s, 0.17 m^3^/s, 0.09 m^3^/s, and 0.06 m^3^/s, respectively. Compared to the SW as the sole source for water replenishment, which, with the optimized water supply scheme, can lead to less total water flow required in each month, its total annual water replenishment requirement can be reduced by 21%. Moreover, the benefits of the optimal replenishment are even more evident from November to April, which was reduced by 33%. This is because the temperature is low and the light intensity is weak in November–April, and the algae growth is slow even if the nutrient concentration increases in the water body replenished with RW; thus, the SD is greatly affected by the SS. The more increased the RW proportion, the lower the SS is, which is more beneficial to the SD—that is, it can better reflect the superiority of the low SS of the RW to improve the SD of urban waters. However, this advantage is not obvious in other months. In conclusion, the findings indicate a maximization of the advantages of the low suspended solids content of the RW to offset the effect on algae growth (caused by its high concentration of nutrients) on the transparency through adjustment of the RW proportion. Consequently, the required inflow for replenishment was reduced, and an optimized supply scheme of RW replenishment was developed. Of course, because the conditions of each water body are different, it needs to be analyzed and confirmed separately.

## 4. Conclusions

The use of RW as a replenishment source for urban waters in water-scarce areas has raised concerns that its high nutrient concentration will increase the nutrient concentration in water bodies, thereby adversely affecting them. Perhaps what the residents most want is to maintain a better water aesthetic quality, but high concentrations of nitrogen and phosphorus in the RW promote algal growth and reduce the aesthetic effect of water bodies. In order to assess the feasibility of RW as a source for water replenishment, water quality modeling was carried out in this study to compare the RW with SW and optimize the water supply scheme of their combined usage from the viewpoint of landscape quality control. By using SD as an indicator, it was found that the negative effect on the landscape quality of algae growth due to the higher nutrient contents of the RW could be offset, to a great extent, by its lower SS concentration than the SW. Partial or complete utilization of RW to replace SW was, thus, proven feasible from the viewpoint of landscape quality control. Furthermore, when using RW as a water replenishment source, algal growth can be inhibited through measures such as aeration, plug flow, and ecological floating beds, thus making greater use of the low SS of the RW to improve the landscape quality of urban waters. However, it should be noted that the SD cannot be the only indicator to assess the feasibility of replacing SW with RW. Other ecological and risk indicators should also be further considered.

## Figures and Tables

**Table 1:** Water quality of SW and RW.

	NH_4_^+^-N	NO_3_^−^-N	IP	SS
SW(mg/L)	1.65	0.35	0.2	60
RW(mg/L)	5	10	0.5	10

**Table 2:** Water quality of influent and dry deposition mass of model boundary conditions.

Month	Water Quality of Influent (mg/L)				Dry Deposition (kg/Month)		
	NH_4_^+^-N	NO_3_^−^-N	IP	SS	NH_4_^+^-N	NO_3_^−^-N	IP
January	0.97	2.89	0.021	12.62	22.7	15.6	3.0
February	0.80	2.95	0.027	7.93	20.5	14.1	2.7
March	0.88	2.86	0.018	12.69	22.2	14.1	2.8
April	0.83	2.51	0.012	13.72	20.4	13.0	2.5
May	0.39	1.66	0.013	16.61	22.1	14.0	2.7
June	0.76	0.50	0.041	11.22	12.6	5.0	1.8
July	0.32	0.53	0.016	14.31	13.3	5.2	1.9
August	0.68	0.87	0.014	12.23	13.5	5.3	1.9
September	0.22	0.66	0.012	13.18	3.8	1.6	0.9
October	0.58	0.97	0.010	15.17	5.7	2.4	1.3
November	1.66	0.78	0.012	9.92	9.4	3.9	2.1
December	1.69	2.06	0.045	11.12	25.4	17.4	3.3

**Table 3:** The main calibrated parameters of the model.

No.	Parameter	Description (Unit)	Value
Hydrodynamic parameters			
1		Resistance	0.035
2		Smagorinsky coefficient	0.28
3		Light refraction index	0.3
Water transparency parameters			
1	γ	Eye’s ability to distinguish contrast	8.9
2	$a_{w}\left(\lambda \right)$	The absorption by pure water (1/m)	0.050
3	$a_{\varphi }\left(\lambda \right)$	The chlorophyll-specific absorption coefficient (m2/mg chl-a)	0.020
4	$a_{p-\varphi }\left(\lambda \right)$	The absorption coefficient by inorganic suspended solids (ISS) (m2/g ISS)	0.08
5	$a_{p-\varphi }^{*}\left(\lambda \right)$	The absorption coefficient by detritus carbon (m2/g DC)	0.24
6	$b_{w}\left(\lambda \right)$	The scattering by pure water (1/m)	0.0019
7	$b_{p-\varphi }\left(\lambda \right)$	The scattering coefficient by ISS (m2/g ISS)	0.025
Water quality parameters			
1	mypc	Growth rate phytoplankton C (per day)	2.8
2	deac	1st order death rate for phytoplankton(per day)	0.10
3	tetg	Temperature dependency growth ratefor phytoplankton	1.14
4	Kn	Half-saturation constant for nitrogen uptake(mg N/L)	0.05
5	Kp	Half-saturation concentration forphosphorus uptake(mg P/L)	0.009
6	kmdm	Detritus C mineralization rate (per day)	0.040

## Data Availability

All the data are available within the manuscript.
